# FastBMD: an online tool for rapid benchmark dose–response analysis of transcriptomics data

**DOI:** 10.1093/bioinformatics/btaa700

**Published:** 2020-08-06

**Authors:** Jessica Ewald, Othman Soufan, Jianguo Xia, Niladri Basu

**Affiliations:** Department of Natural Resource Sciences, McGill University, Montreal, QC H9X 3V9, Canada; Institute of Parasitology, McGill University, Montreal, QC H9X 3V9, Canada; Institute of Parasitology, McGill University, Montreal, QC H9X 3V9, Canada; Department of Natural Resource Sciences, McGill University, Montreal, QC H9X 3V9, Canada

## Abstract

**Motivation:**

Transcriptomics dose–response analysis is a promising new approach method for toxicity testing. While international regulatory agencies have spent substantial effort establishing a standardized statistical approach, existing software that follows this approach is computationally inefficient and must be locally installed.

**Results:**

FastBMD is a web-based tool that implements standardized methods for transcriptomics benchmark dose–response analysis in R. It is >60 times faster than the current leading software, supports transcriptomics data from 13 species, and offers a comprehensive analytical pipeline that goes from processing and normalization of raw gene expression values to interactive exploration of pathway-level benchmark dose results.

**Availability and implementation:**

FastBMD is freely available at www.fastbmd.ca.

**Supplementary information:**

Supplementary data are available at *Bioinformatics* online.

## 1 Introduction

Dose–response analysis (DRA) is a classic method in toxicology used to identify the dose of a chemical that causes a predetermined change in a physiological response; traditionally, this would be an apical outcome such as mortality. This identified dose, called the benchmark dose (BMD), is a key component for developing regulatory standards that aim to protect human and ecological health from adverse effects associated with exposure to chemicals.

BMDs derived from transcriptomics data from short-term exposures are similar to BMDs derived from apical outcomes from long-term exposures (Thomas *et al.*, 2013). Thus, there has recently been a concerted effort by regulatory agencies, including the U.S. National Toxicology Program (NTP) and Health Canada, to develop standardized methods for DRA with transcriptomics data (Farmahin *et al.*, 2017; US NTP, 2018, Supplementary Table S1). The leading tool, BMDExpress, is a Java-based software that follows these standardized transcriptomics DRA methods (Phillips *et al.*, 2019). However, it is computationally intensive, requires local installation, and is not easily integrated with the wealth of bioinformatics resources available in R.

Here, we present FastBMD, a computationally efficient implementation of the NTP’s approach to transcriptomics DRA (US NTP, 2018). FastBMD is a freely available web-based software (www.fastbmd.ca) that supports BMD analysis at the gene, pathway, and transcriptomic levels. FastBMD currently supports microarray and RNA-sequencing transcriptomics data from 13 species (Supplementary Table S2), and also includes an annotation-free pipeline for gene and transcriptomic-level BMD analysis for non-model organisms.

## 2 Implementation

Previous work demonstrated that core non-linear curve fitting algorithms can be efficiently implemented in R (Larras *et al.*, 2018; Ritz *et al.*, 2015). This provided the foundation for FastBMD as an R-based DRA software that follows the NTP approach (US NTP, 2018).

**Fig. 1.**
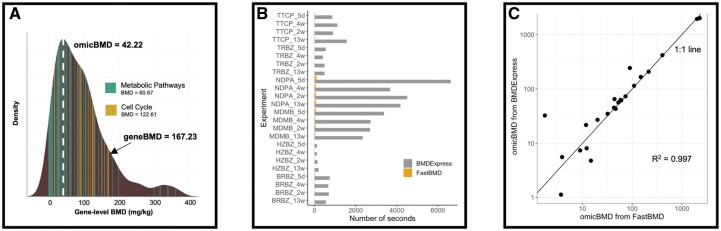
FastBMD performance. (**A**) Snapshot of interactive gene, pathway and transcriptomic BMD visualization in FastBMD for the TRBZ (13 weeks) dataset. (**B**) Comparison of elapsed time during curve-fitting between FastBMD (yellow) and BMDExpress (grey) for 24 datasets. (**C**) OmicBMDs from FastBMD and BMDExpress for 24 datasets.

Prior to curve fitting, a matrix of expression values is annotated (Supplementary Table S2), summarized at the gene level, filtered according to abundance and variance thresholds, and normalized. Genes are filtered according to fold-change and *P*-value thresholds to eliminate those that are unlikely to have dose-dependent behaviour prior to the computationally intensive curve fitting step. For DRA, model parameters are found for up to 10 statistical models using base R non-linear search functions (Larras *et al.*, 2018; Ritz *et al.*, 2015), and filtered with a lack-of-fit *P*-value threshold (US NTP, 2018). The best-fit model is selected for each gene. The gene-level BMD (geneBMD) and its upper and lower 95% confidence intervals are calculated using the likelihood-profiling method based on the mean and standard deviation of control expression values (Ritz *et al.*, 2015; US NTP, 2018). GeneBMDs are filtered to remove any that occur above the highest measured dose, or that have a wide confidence interval (US NTP, 2018). The transcriptomic-level (omicBMD) is calculated using the distribution of geneBMDs (Pagé-Larivière *et al.*, 2019). Pathway-level BMDs are calculated as the bootstrapped median of geneBMDs within each pathway. Finally, the gene, pathway, and transcriptomic-level results are integrated with interactive plots and tables (Fig. 1A). Detailed explanations of each component of the analysis can be found in the ‘FAQ’ and ‘Resources’ tabs on www.fastbmd.ca.

FastBMD was implemented based on the PrimeFaces (v8.0) component library (http://primefaces.org/) and R (version 3.6.2). The interactive features were developed using Plotly.js (https://plot.ly). The system is hosted on a Google Cloud instance (12 virtual CPUs, 70 GB RAM), except for curve fitting which is computed in parallel through a microservice hosted on a dedicated server (24 cores, 128 GB RAM) using SpringBoot.

## 3 Materials and methods

The performance of FastBMD was compared to that of BMDExpress 2 with microarray data measured in adult rats from 24 separate dose–response experiments (Supplementary Table S3) (Thomas *et al.*, 2013). Data were quantile normalized in R, and then analysed using both FastBMD and BMDExpress on a Macbook Pro with 4 cores and 8 GB RAM. Genes that did not have a fold change of at least two for any dose group compared to the control were filtered out. All statistical models other than 3° and 4° polynomials were fit to the expression of each remaining gene. More methodological details are given in Supplementary Materials.

## 4 Results and discussion

FastBMD was over 60 times faster than BMDExpress, taking 0.18 h to compute geneBMDs for all 24 experiments compared to 11.03 h for BMDExpress (Fig. 1B). The omicBMD^mode^ from each experiment were extremely similar with an even distribution around the 1:1 line and an *R*^2^ of 0.997 (Fig. 1C). Sources of variation in the results between the two softwares are discussed in Supplementary Materials. The increased efficiency can be mainly explained by the curve fitting algorithm. In FastBMD, parameters are roughly estimated using the input data, and then fed into a modern non-linear parameter search algorithm that quickly either converges or fails. In contrast, BMDExpress searches the parameter space until either a solution is found, or the user-specified timeout period is surpassed.

FastBMD is designed to be a flexible tool that can accommodate diverse transcriptomics data. It addresses reproducibility by allowing users to download results after each analytical step and generate summary reports. Since it is implemented in R, FastBMD is uniquely positioned to leverage existing statistical packages to rapidly implement future updates as the regulatory and scientific communities continue to refine the recommended approach. In the future, we plan to incorporate knowledgebases of environmental chemical concentrations, expanded gene-set libraries and baseline gene expression levels in common target tissues to provide additional context for the BMD results.

## Funding

This work was supported by Canada’s Natural Sciences and Engineering Research Council (Discovery Grants to J.X. and N.B.; Canada Graduate Scholarship to J.E.), and the Canada Research Chairs Program (to J.X. and N.B.). The authors also thank Jason O’Brien, Florence Pagé-Larivière, Doug Crump, and other members of the EcoToxChip project (funded by Genome Canada) for their advice.

*Conflict of Interest*: none declared.

## Supplementary Material

btaa700_Supplementary_Data
