# Supplementary material for: FastBMD: an online tool for rapid benchmark dose–response analysis of transcriptomics data
Source: Bioinformatics. 2020 Aug 6;37(7):1035–6. doi: 10.1093/bioinformatics/btaa700 (PMC8128449; doi:10.1093/bioinformatics/btaa700)
Supplement: btaa700_Supplementary_Data [file btaa700_supplementary_data.doc]

**Supplementary Materials**

**SM Table 1: Comparison of FastBMD to popular dose-response software.**

**SM Table 2: Species and annotation ID types supported by FastBMD.**

**SM Table 3: Description of dose-response experiments used to test FastBMD performance.**

**SM Figure 1: Number of probes that passed each filter.**

**SM Figure 2: Comparison of the omicBMDs from FastBMD and BMDExpress**

**SM Figure 3: Overlapped and unique probes with geneBMDs.**

**SM Figure 4: Probe-level results from FastBMD and BMDExpress.**

**SM Table 1: Comparison of FastBMD to popular dose-response software.** The platform type impacts accessibility and flexibility, with web platforms being the most accessible and R being the most flexible. Speed is estimated relative to the other software, with one check representing the slowest and three being the fastest. See Figure 1 for a more detailed speed comparison with BMDExpress 2. “Batch analysis” refers to the ability to upload and process multiple transcriptomics dose-response matrices at one time.

| **Software** | **FastBMD** | **BMDExpress 2** | **BMDS 3** | **PROAST** | **DROmics** |
| --- | --- | --- | --- | --- | --- |
| **Platform** | Web | Locally installed** | Locally installed*** | R and Web | R and Web |
| **Speed** | 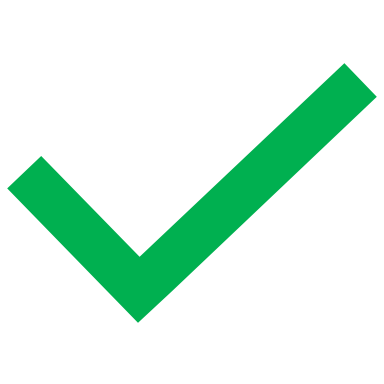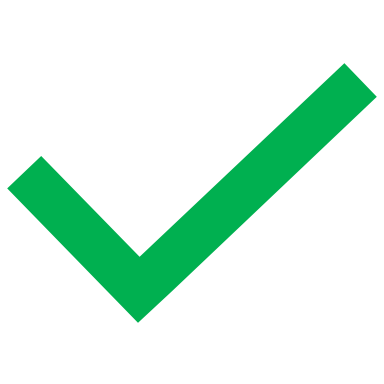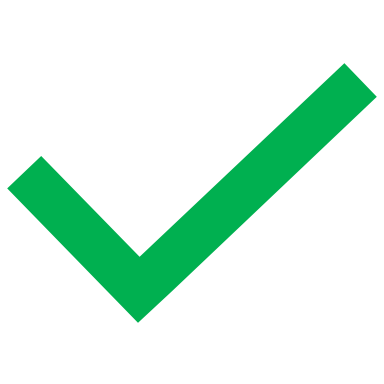 | 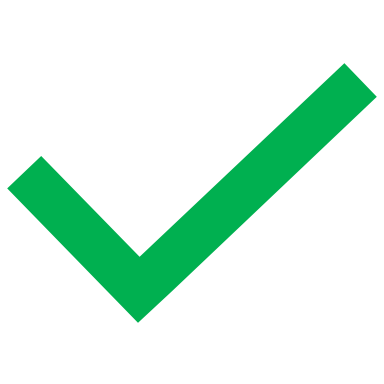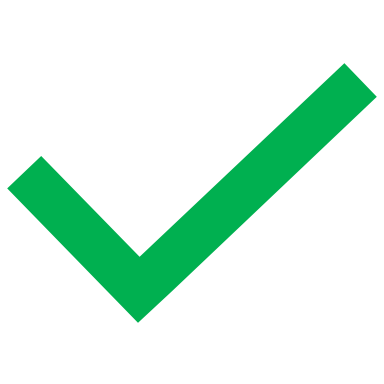 | 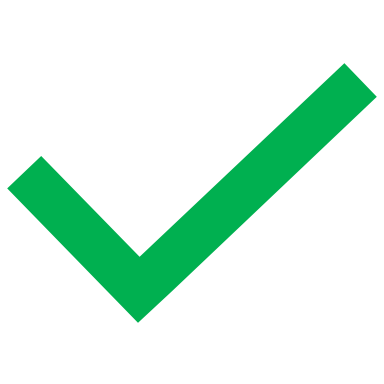* | R: 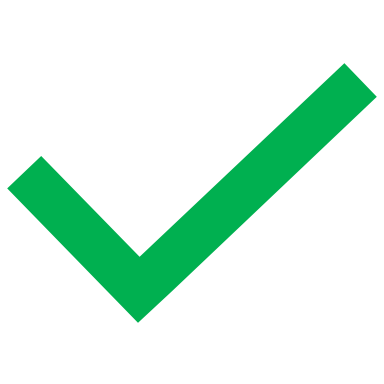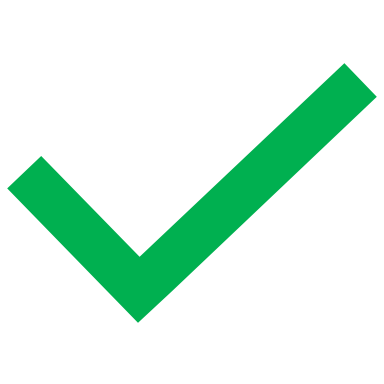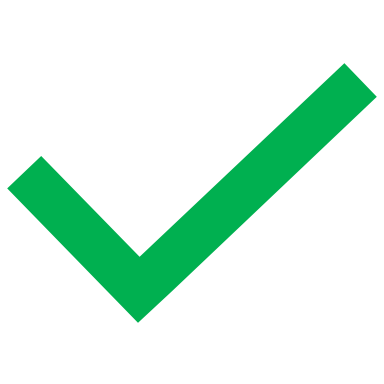*  Web: 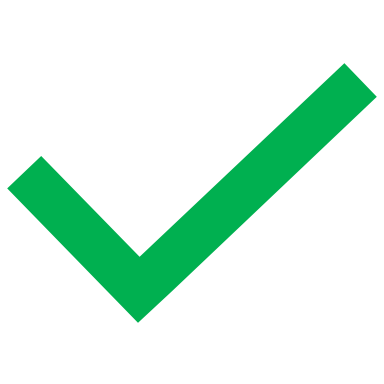* | R: 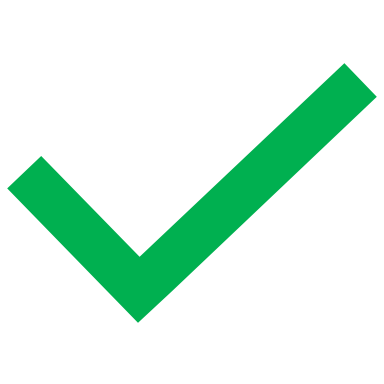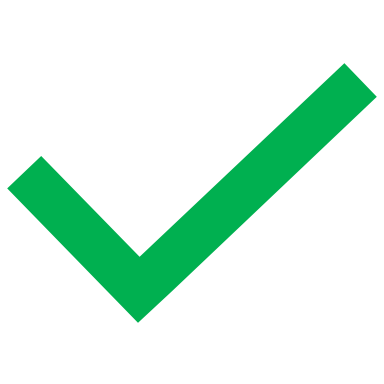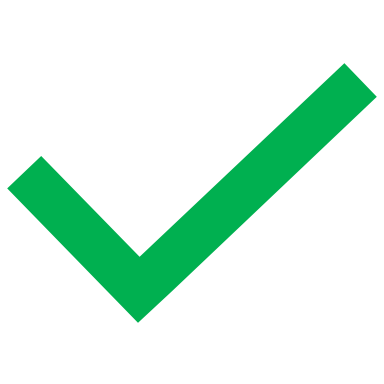  Web: 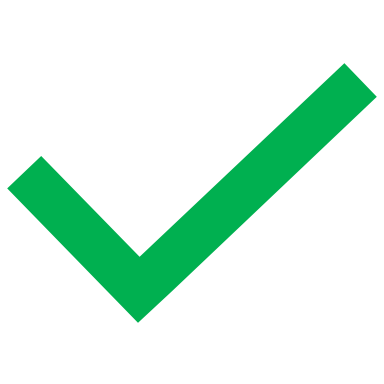 |
| **Number of continuous models** | 10 | 10 | 10 | 47 | 7 |
| **Interactive data visualization** | Yes | Yes | No | No | No |
| **Designed for transcriptomics** | Yes | Yes | No | No | Yes |
| **Supports batch analysis** | No | Yes | No | No | No |
| **Supports gene set analysis** | Yes | Yes | NA | NA | No |
| **Follows NTP approach** | Yes | Yes | NA | NA | No |
| **Citation** | NA | (Phillips *et al.*, 2019) | (Davis *et al.*, 2011) | (Hardy *et al.*, 2017) | (Larras *et al.*, 2018) |

*: This software is not purposefully designed for transcriptomics data, thus additional code must be written by the user to automate curve fitting for 100s – 1000s of genes. The time it would take to develop this additional code is not considered in the speed ranking.

**: BMDExpress 2.0 is compatible with Windows, Mac OSX, and Linux operating systems.

***: BMDS 3.0 is compatible with Windows operating systems. Microsoft Excel must also be installed.

**SM Table 2: Species and annotation ID types supported by FastBMD.** Specific supported microarray platforms can be found by visiting [www.fastbmd.ca](http://www.fastbmd.ca), choosing the organism of interest from the “Specify Organism” dropdown menu, and then consulting the “ID type” dropdown menu. Ens. G = Ensembl Gene; Ens. P = Ensembl Protein; Ens. T = Ensembl Transcript; OGS = Official Gene Symbol; ORF = Open Reading Frame.

| Species | Entrez | Refseq | Ens. G | Ens. P | Ens. T | OGS | Genbank | Uniprot | String | Other |
| --- | --- | --- | --- | --- | --- | --- | --- | --- | --- | --- |
| *A. thaliana*  (Arabidopsis) | X | X | X | X | X | X | X | X |  | Tair |
| *B. taurus*  (cow) | X | X | X | X | X | X | X | X |  |  |
| *C. elegans*  (roundworm) | X | X | X | X | X | X | X | X | X | Wormbase |
| *C. japonica*  (Japanese quail) | X | X | X | X | X | X |  |  |  |  |
| *D. melanogaster*  (fruit fly) | X | X | X | X | X | X | X | X | X | Flybase |
| *D. rerio*  (zebrafish) | X | X | X | X | X | X | X | X | X | 1 microarray |
| *G. gallus*  (chicken) | X | X | X | X | X | X |  |  |  |  |
| *H. sapiens*  (human) | X | X | X | X | X | X | X | X |  | 22 microarrays |
| *M. musculus*  (mouse) | X | X | X | X | X | X | X | X | X | 19 microarrays |
| *R. norvegicus*  (rat) | X | X | X | X | X | X | X | X |  | 4 microarrays |
| *S. cerevisae*  (yeast) | X | X | X | X | X | X |  | X | X | ORF identifiers |
| *S. scrofa*  (pig) | X | X | X | X | X | X | X | X |  |  |
| *X. laevis*  (African clawed frog) | X | X |  |  |  |  |  |  |  |  |

**Comparison between FastBMD and BMDExpress 2**

*Datasets*

The performance of FastBMD was compared to that of BMDExpress 2 (Phillips *et al.*, 2019) by analyzing 24 previously published microarray dose-response datasets (Thomas *et al.*, 2013) with both software. The transcriptomic data were measured in adult rats (*Rattus norvegicus*) that were exposed to five doses of six chemicals for four different exposure durations (five days, two weeks, four weeks, thirteen weeks) with Affymetrix HT Rat230+ PM microarrays. This resulted in 24 distinct datasets, as described in SM Table 3. Data were downloaded from NCBI’s Gene Expression Omnibus (accession = GSE45892). More details on the exposures can be found in the original publication (Thomas *et al.*, 2013).

**SM Table 3: Description of dose-response experiments used to test FastBMD performance.** Exposures that generated the previously published datasets used to compare the performance of FastBMD to BMDExpress. For dose units: mkd = mg per kg per day; ppm = parts per million.

| Chemical | Doses | tissue |
| --- | --- | --- |
| 1,2,4-Tribromobenzene  (TRBZ) | 0, 2.5, 5, 10, 25, 75 mkd | Liver |
| Bromobenzene  (BRBZ) | 0, 25, 100, 200, 300, 400 mkd | Liver |
| 2,3,4,6-Tetrachlorophenol  (TTCP) | 0, 10, 25, 50, 100, 200 mkd | Liver |
| 4,4’-Methylenebis(*N,N*-dimethyl) benzenamine  (MDMB) | 0, 50, 200, 375, 500, 750 ppm | Thyroid |
| N-Nitrosodiphenylamine  (NDPA) | 0, 250, 1000, 2000, 3000, 4000 ppm | Bladder |
| Hydrazobenzene  (HZBZ) | 0, 5, 20, 80, 200, 300 ppm | Liver |

*Statistical analysis*

Prior to dose-response analysis, each dataset was quantile normalized in R using the ‘limma’ R package (Ritchie *et al.,* 2015). FastBMD was run offline on the same computer and using the same computational resources as BMDExpress so that the elapsed time for each analysis could be directly compared between the two software. Within both software, each of the 24 datasets were filtered to remove any probe that did not have a fold-change of greater than two for any dose group. For the BMD analysis, all models except for the higher order polynomials (Exp2, Exp3, Exp4, Exp5, Linear, Poly2, Hill, and Power models) were fit to the expression values of each probe. These model fits were then used to calculate the geneBMDs and their 95% upper (geneBMD_u_) and lower (geneBMD_l_) confidence intervals.

For BMD analysis in BMDExpress, the following parameters were selected:

- Maximum Iterations: 250
- Confidence Level: 0.95
- Constant Variance: TRUE
- BMR Type: Standard Deviation
- BMR Factor: 1 SD
- Restrict Power: >= 1
- BMDL and BMDU: Compute but ignore non-convergence in best model selection
- Best Poly Model Test: Lowest AIC
- P-value Cutoff: 0.1
- Number of threads: 8
- Model Execution Timeout (secs): 30

For BMD analysis in FastBMD, the following parameters were selected:

- Lack-of-fit p-value: 0.1
- BMR Factor: 1 SD
- Control expression: Evaluate model at 0

After gene-level BMD analysis, model fits and geneBMD results were downloaded from both software and imported into R. These results were filtered to remove any probes where the geneBMD was greater than the highest dose, or where the geneBMD_u_/geneBMD_l_ was greater than 40. The remaining geneBMDs were used to compute an omicBMD for each dose-response experiment using the “mode” method. Curve fitting results were compared between FastBMD and BMDExpress based on the time to compute model fits and geneBMDs, number of probes that passed each filtering step, omicBMD values, geneBMD values, distribution of best fit models, and model fit quality. Model fit quality was assessed using Akaike’s Information Criterion (AIC), which is a modified measure of a model’s prediction error that applies increasingly large penalties for more complex models. Thus, a model that has a smaller prediction error, but many parameters, may have a worse (higher) AIC score than a simpler model that has a slightly larger prediction error.

Comparing AIC scores across BMDExpress and FastBMD models was challenging as the AIC values returned by BMDExpress and FastBMD were different even when the model had the exact same coefficients. Thus, AIC values had to be re-computed in R using the same method for both software, however there is no straightforward method to manually create model fit objects in R with previously defined coefficients. To overcome this, we re-ran “nls”, a non-linear model fitting algorithm in base R, for each model fit. Using the previously found coefficients as starting values and restricting the number of iterations forced “nls” to converge on the coefficients from FastBMD and BMDExpress, returning model fit objects that could be used to compute the AIC.

*Results and Discussion*

Some datasets exhibited a much stronger dose-dependent response to chemical exposure than others, resulting in different numbers of probes that passed the fold change, curve fitting, and BMD filters across the 24 datasets (SM Figure 1). The number of geneBMDs computed for each dataset ranged from a minimum of 16 (TTCP – 4 weeks, BMDExpress) to a maximum of 2945 (NDPA – 5 days, FastBMD). In general, the omicBMDs from both software were similar to each other, with an R^2^ of 0.997 (SM Figure 2a). OmicBMDs were more variable between the two software when there were smaller numbers of geneBMDs (Figure 2b). This makes sense as an omicBMD that is computed from 10s of geneBMDs likely has more associated uncertainty than one that is computed from 100s or 1000s of geneBMDs.


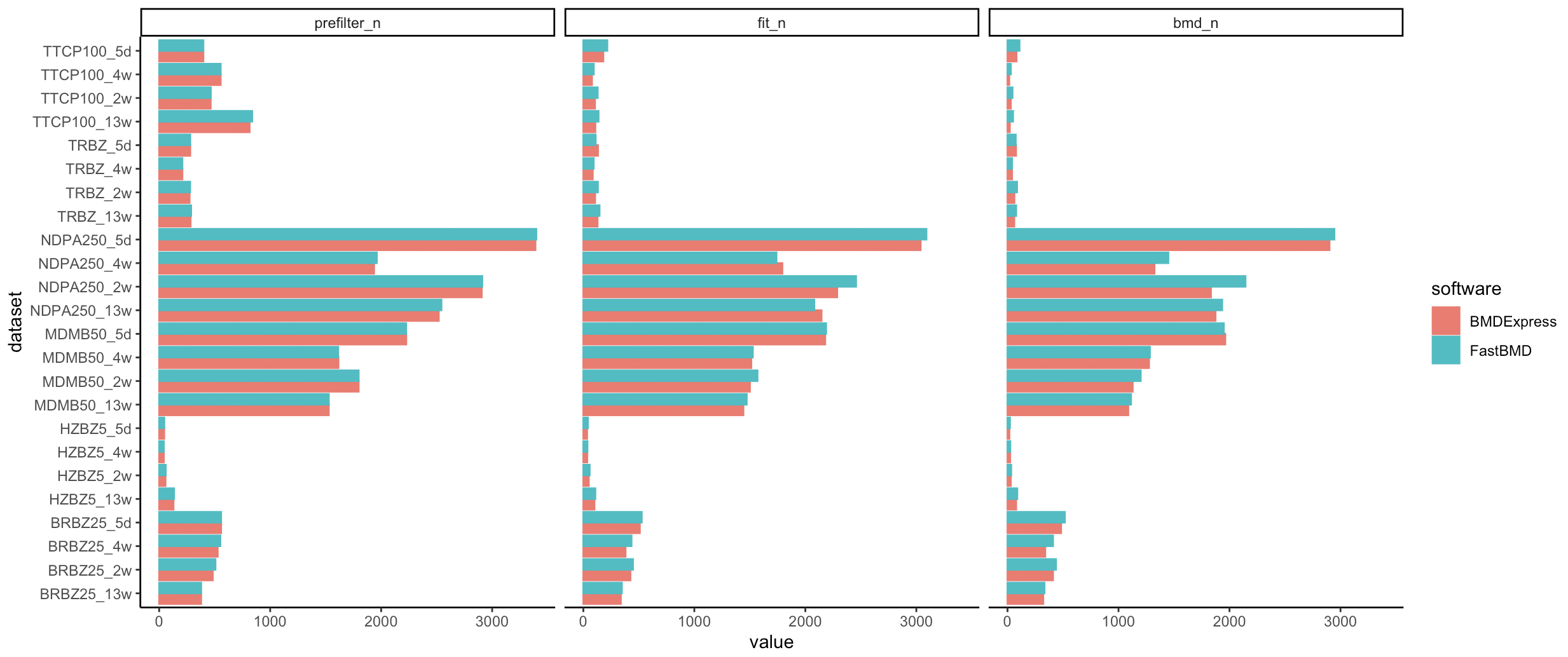


**SM Figure 1: Number of probes that passed each filter.** Bars show the number of probes that passed each of the filtering steps. (blue = FastBMD; pink = BMDExpress; prefilter_n = after fold-change filter; fit_n = after lack-of-fit p-value filter; bmd_n = after BMD quality filters).


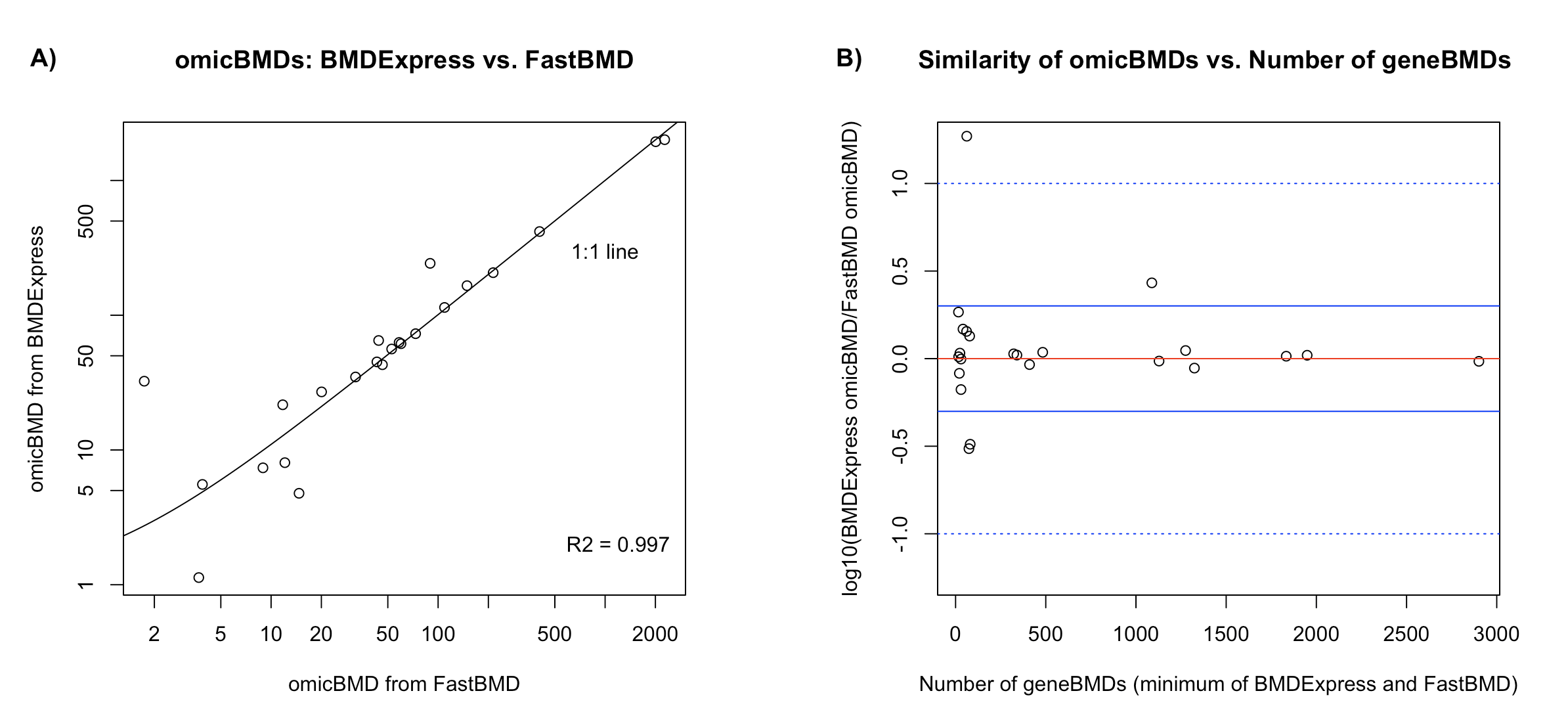


**SM Figure 2: Comparison of the omicBMDs from FastBMD and BMDExpress.** A) omicBMDs computed with the “mode” method using both FastBMD and BMDExpress for the datasets summarized in SM Table 2. B) log10 of the ratio of BMDExpress:FastBMD omicBMDs plotted against the minimum number of geneBMDs used to compute the omicBMD from both software. Given that the y-axis is on a log10 scale, the solid red line indicates a fold-change of 1 (no change), the solid blue line is a fold-change of 2, and the dotted blue line is a fold-change of 10.

Across all 24 experiments, BMDExpress and FastBMD found 15 479 and 16 394 geneBMDs respectively. The majority of the fits were for the same probes (*n* = 14 598), however BMDExpress and FastBMD both found model fits that passed all of the quality criteria for some probes (BMDExpress: *n* = 881; FastBMD: *n* = 1796) that the other software did not find (SM Figure 3a). For the models fit to the unique probes in particular, there was a different distribution of best-fitting model types, with BMDExpress tending to find more Hill and Power model fits and FastBMD finding more Exponential and Polynomial fits (SM Figure 3b). This shows that while neither curve fitting algorithm is the best choice in all scenarios, the FastBMD algorithm does find high-quality model fits more often than the BMDExpress algorithm does.


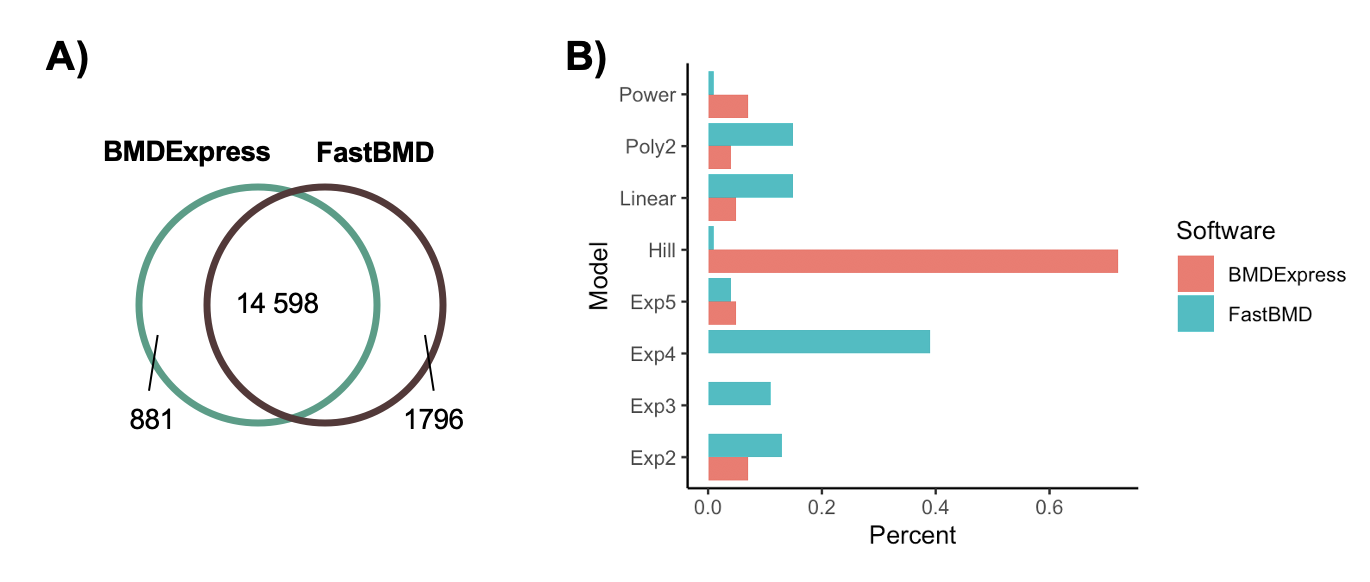


**SM Figure 3: Overlapped and unique probes with geneBMDs.** A) The overlap of probes with geneBMDs that passed all quality filters from BMDExpress and FastBMD. B) Percent of best fit models of each type that had a geneBMD from one software but not the other (*n* = 881 for BMDExpress; *n* = 1796 for FastBMD).

The AIC scores were re-computed for the models that were fit to the same probes by both software. Overall, AIC scores were able to be re-computed for both the FastBMD and BMDExpress model fits for 13 224 out of the 14 598 shared probes (90.6%). AIC scores were unable to be computed for all genes since forcing ‘nls’ to rerun with certain restrictions caused errors for some data, while loosening the restrictions had the effect of allowing ‘nls’ to find new model parameters. Errors were thrown by both FastBMD and BMDExpress models, so we are working under the assumption that the re-computed AIC values for 90.6% of probes are representative of the full set of models.

The re-computed AIC values were very similar across all probes (SM Figure 4a). Out of the 13 224 probes with AIC values for both software, 10 080 had the exact same individual parameters and model types. They had nearly identical BMDs, except for 18 of the Poly2 fits (SM Figure 4b). This can be explained by how each software computes the benchmark response (BMR). The benchmark response (BMR) is found by evaluating the fitted model at zero, and then either adding or subtracting the standard deviation of the residuals depending on whether the adverse direction is positive or negative. In FastBMD, the adverse direction is determined by fitting a linear model to the expression values for each probe and seeing if the slope coefficient is positive or negative. Since Poly2 curves can change direction, it’s possible for the overall slope to be in the opposite direction as when the curve first surpasses one standard deviation of the residuals. Thus, on rare occasions (1.4% of Poly2 fits; 18/1282 occurrences), BMDExpress and FastBMD can return substantially different gene-level BMDs from the same Poly2 fits.

Probes with different models (*n* = 3144) produced geneBMDs that were less consistent across the two software (SM Figure 4c). The vast majority of these probes (99%) had different best-fitting model types, which then returned less similar geneBMDs for the same probe expression values. To determine which software tended to find models with higher quality fits, the differences between FastBMD and BMDExpress AIC scores were visualized with a density plot (SM Figure 4d). The differences were negative 59% of the time, indicating that when the software found different models, FastBMD found higher quality fits more often than BMDExpress, although neither algorithm performed the best in all scenarios.


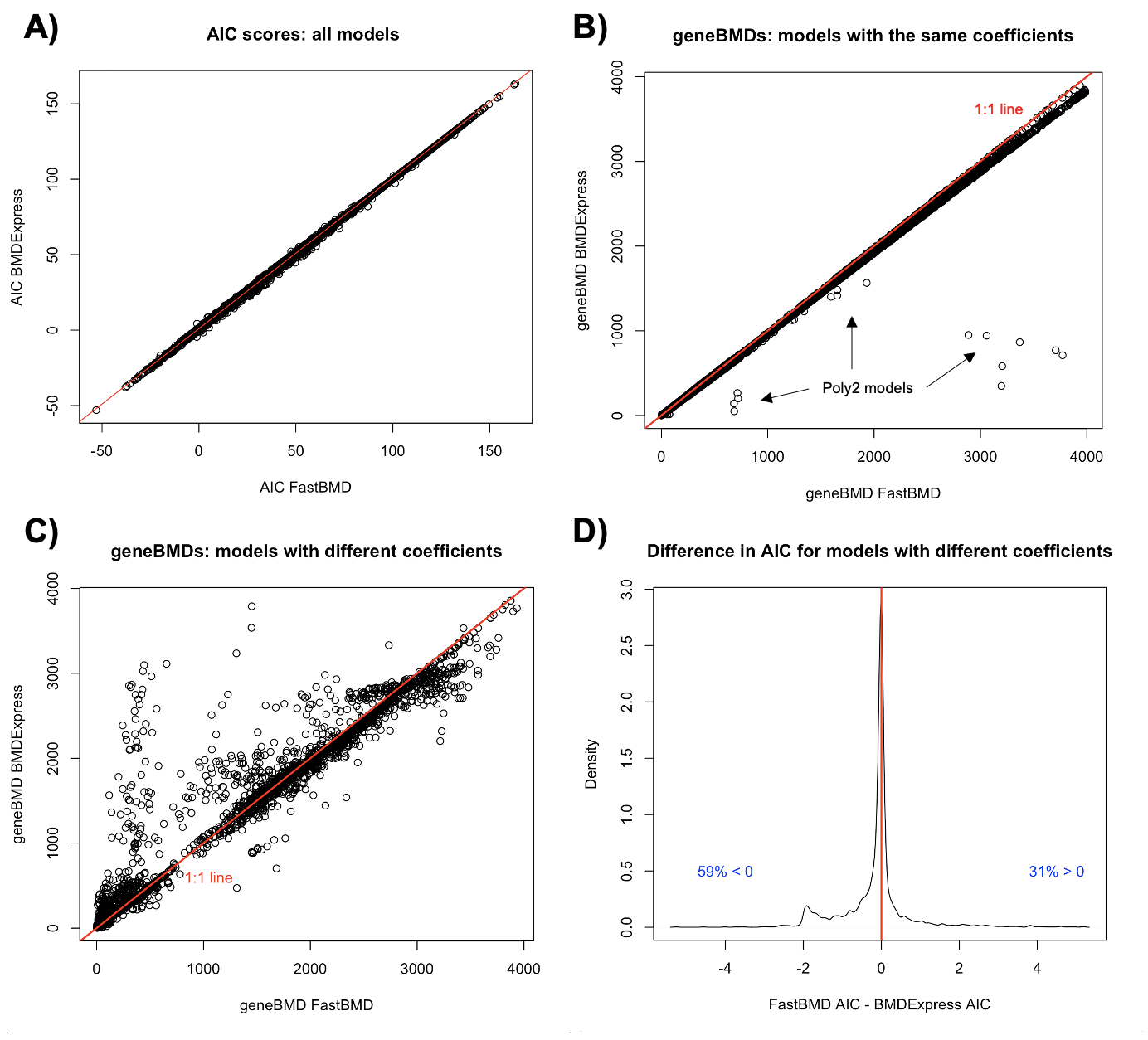


**SM Figure 4: Probe-level results from FastBMD and BMDExpress.** A) AIC scores for models returned by FastBMD and BMDExpress (*n* = 13 224). B) geneBMD values for models with the same type and coefficients from FastBMD and BMDExpress that also have AIC scores (*n* = 10 080). Upon investigation, all noticeable outliers are from Poly2 model fits (*n* = 18 Poly2 outliers). C) geneBMD vaues for models with different coefficients and/or types (*n* = 3144). D) Distribution of differences between FastBMD and BMDExpress AIC values from models with different coefficients and/or types (*n* = 3144). Negative and positive differences indicate that the FastBMD and BMDExpress models had a higher quality fit, respectively.

The majority of the variation in geneBMDs appears to be from scenarios where BMDExpress and FastBMD find different best-fitting model types (SM Figure 4c), even though these model fits still had very similar AIC values (SM Figure 4a). It is concerning that two models with almost the same quality fit can produce substantially different BMDs; this may be an inherent limitation to using parametric models for BMD analysis. When there were differences in the best-fitting model type, FastBMD tended to find higher quality model fits more often than BMDExpress (SM Figure 3a, SM Figure 4d), although BMDExpress does perform slightly better at computing BMDs from Poly2 fits.

BMDExpress and FastBMD both implement the modeling philosophy that is outlined in the NTP approach to transcriptomic dose-response modeling. Even though the NTP recommendations are quite detailed, there are still practical design choices that must be made when implementing the statistical workflow, for example which non-linear parameter search algorithm to choose or how to break ties between model fit AIC scores. For many of these decisions, there isn’t a clear choice that performs best in all scenarios. However, despite small implementation differences like these, FastBMD and BMDExpress returned the exact same best-fit model for 76% of fitted models. When considering both the number of times that both software did not find any model and when the software returned the same model across all 24 datasets, FastBMD and BMDExpress produced the same results for >99% of the probes.

**References**

Phillips JR, Svoboda DL, Tandon A, Patel S, Sedykh A, Mav D, Kuo B, Yauk CL, Yang L, Thomas RS. “BMDExpress 2: enhanced transcriptomic dose-response analysis workflow.” *Bioinformatics* 2019, 35:1780-1782.

Ritchie ME, Phipson B, Wu D, Hu Y, Law CW, Shi W, Smyth GK. “limma powers differential expression analyses for RNA-sequencing and microarray studies.” *Nucleic Acids Research* 2015, 43:e47-e47.

Thomas RS, Wesselkamper SC, Wang NCY, Zhao QJ, Petersen DD, Lambert JC, Cote I, Yang L, Healy E, Black MB, Clewell HJ, Allen BC, Andersen ME. "Temporal concordance between apical and transcriptional points of departure for chemical risk assessment." *Toxicological Sciences* 2013, 134.1: 180-194.

Allen DJ, Gift JS, and Zhao QJ. "Introduction to benchmark dose methods and US EPA's benchmark dose software (BMDS) version 2.1.1." *Toxicology and Applied Pharmacology* 2011, 254.2: 181-191.

Larras F, Billoir E, Baillard V, Siberchicot A, Scholz S, Wubet T, Tarkka M, Schmitt-Jansen M, Delignette-Muller M. "DRomics: A turnkey tool to support the use of the dose–response framework for Omics data in ecological risk assessment." *Environmental Science & Technology* 2018, 52.24: 14461-14468.

EFSA Scientific Committees, Hardy A, Benford D, Halldorsson D, Jeger MJ, Knutsen KH, More S, Mortensen A, Naegeli H, Noteborn H, Ockleford C, Ricci A, Rychen G, Silano V, Solecki R, Turck D, Aerts M, Bodin L, Davis A, Edler L, Gundert‐Remy U, Sand S, Slob W, Bottex B, Abrahantes JC, Marques DC, Kass G, Schlatter JR. "Update: use of the benchmark dose approach in risk assessment.” *EFSA Journal* 2017.
